# Items

**Published:** 1882-12

**Authors:** 


### Items.

METEOROLOGICAL DATA.—For the month of Oct., 1882. H. C. Smyth, Observer, U. S. Signal Station, Atlanta, Ga.

| | | |
|---|---|---|
| BAROMETER | —Mean | 30.090 |
| | Highest | 30.302 on 5th. |
| | Lowest | 29.815 on 11th. |
| | Range | .487 |
| THERMOMETER | —Mean | 65.0 |
| | Highest | 79.8 on 1st. |
| | Lowest | 46.2 on 24th. |
| | Range | 33.6 |
| | Greatest Daily Range | 21.2 on 17, 25. |
| | Least Daily Range | 6.0 on 20th. |
| Humidity—Mean | | 73.5 |
| Dew-point—Mean | | 55.0 |
| Rainfall—Total | | 1.35 inches. |
| | Greatest Daily | .55 on 19th. |
| | Total Number of Days | 7 |
| Wind—Prevailing Direction | | N. E. |
| | Maximum Velocity | E., 22 miles. |

---

THE disgraceful practice truthfully referred to in the accompanying extract from the *Medical Press and Circular* is a wide-spread evil. It applies to many medical men in this country who assume to be reputable representatives of the profession, but who use their association with it for selfish ends only, and who degrade it by their acts and by their alliance with it:

"Dr. ——, of No. —— Square, Dublin, Fellow and Examiner (*sic*) of the Royal College of Surgeons, the very eminent and consulting surgeon of Ireland, paid a visit to —— yesterday, and was much pleased with what he saw of our city, particularly with our noble river, which that excellent authority considers ought to keep us free from epidemic disease. Surgeon —— returned to Dublin this day."

"The ill grammar and errors of fact of this paragraph make it obvious that it was not written by the gentleman in question; but we feel it necessary to say that such a professional puff is indecent, and certainly not calculated to maintain the reputation, honor and dignity of any person or institution. It needs to be at once publicly and sternly repudiated. Reporters are men of business. They print these paragraphs because they think that their doing so will be agreeable to the person referred to; and they have some justification for that impression when they see every trip for a day out of town "by telegram" chronicled in the fashion columns of the papers. Those who suffer as the surgeon here referred to has done owe it to the profession that they shall at once make the public aware that they utterly repudiate such puffs, and that surgeons of respectability do not descend to such means of achieving publicity."

---

Recently we had the pleasure of presenting to the notice of the profession a paper by Dr. George H. Noble, of this city, descriptive of a novel apparatus for the abortive treatment of mammary abscess.

The writer claimed for it simplicity, cheapness and efficiency—which claims the originator sustained by the report of a number of cases in which its merits had been fairly tested.

We are informed by a correspondent in New York that he had seen the apparatus—which is essentially a three-tailed bandage—applied and eulogized at some length, by the Professor of Gynecology, at a clinic in Bellevue Hospital Medical College. Our correspondent adds that the aforesaid professor "did not acknowledge his authority, except to say that he had recently seen an account of it in some journal."

We have known of this professor as the brilliant lecturer on physiology at Boston, and as the esteemed successor of the elegant and lamented Elliott at Bellevue, and have never observed that he was deficient in courtesy; but we respectfully submit, if the utility of the appliance so strongly commended itself to his judgment, that a mere verbal acknowledgment of its author would have been proper and right, if not as an act of courtesy, certainly as a good example to his pupils. The fact that the gentleman whose suggestion the professor adopted is not a famous metropolitan practitioner does not alter the case and does not impair his just claim to deserved recognition.

---

In an editorial, condemning the practice of medical diploma peddling, the *Philadelphia Medical Times* exclaims: "Legislation has not done enough. The editorial war must still go on until it is recognized that medical colleges should be educational institutions merely, not peddlers of the right to vex, worry, and destroy a long-suffering community that has so far endured with stupid patience, but which, like the beast of Balaam, may finally find a voice.

"We hail with delight every new birth of a medical college, as a further addition to the burden which will only be laid aside when its weight grows so heavy that it can no longer be borne."

That happy day, should it ever come, will be hailed with joy throughout this sorely surfeited country.

---

We find in the *Chicago Medical Journal and Examiner* the subjoined interesting historical data relating to German medical colleges. By contrast, some of our own hopeful institutions seem to be profoundly afflicted with youngness. The university of Prague was founded anno Dei

1348; Vienna 1365; Heidelberg 1386; Leipzig 1409; Freiburg 1454; Greifswald 1456; Basel 1460; Munich 1472; Jena 1558; Wuerzburg 1582; Gottingen 1737; Berlin 1810; Zurich 1833. Wuerzburg is celebrating this year its third centenary jubilee.

---

THE *College and Clinical Record* publishes the following anecdote of Jenner: The celebrated Dr. Jenner, who introduced vaccination, was a man of genial wit, and the following lines addressed to a lady upon the recovery of her daughter, and sent with a pair of ducks, affords a specimen of his facetious vein:—

"I've despatched, my dear madam, this scrap of a letter,
To say that Miss ——— is very much better;
A regular doctor no longer she lacks,
And therefore I've sent her a couple of quacks."

---

IT is suggested by M. Tarnier, a Paris surgeon, to keep puny infants in an incubator, similar to the incubators used for poultry, for two days to six weeks after birth.

His experiments upon a considerable number of six, seven and eight months children have been very encouraging, and he believes that many lives among the prematurely born infants may be saved by the employment, in this manner, of artificial heat.

---

THE Cincinnati *Lancet and Clinic* says of nitrite of amyl, that an item has been going the rounds of the medical press recommending this agent as a remedy to arrest chill in cases of ague. We tried it, almost killed the patient and did not stop the rigor. It is too powerful and dangerous a remedy to use for such a purpose.

---

AN Indiana court granted a divorce upon the prayer of a woman, the wife of a physician doing a large and lucrative business, based upon his refusal to give up his night practice.

The presiding judge praised the progressive liberality of the laws which permitted the release!

